# The Bifixation Field as a Function of Viewing Distance

**DOI:** 10.1155/2014/274803

**Published:** 2014-05-28

**Authors:** Philip M. Grove, Alistair P. Mapp, Hiroshi Ono

**Affiliations:** ^1^School of Psychology, The University of Queensland, St. Lucia, QLD 4072, Australia; ^2^Centre for Vision Research and Department of Psychology, York University, Toronto, ON, Canada M3J 1P3

## Abstract

Hering reported that the area over which he could bifixate a target was smaller at near convergence distances than far convergence distances and predicted that in extreme horizontal gaze positions, the temporally directed eye lags behind the nasally directed eye. We tested these predictions using a subjective index of eye position. Experiment  1 confirmed that the bifixation field was significantly smaller at near convergence distances. When bifixation broke down at the near distance, the nasally directed eye lagged behind the temporally directed eye for all observers. At the far distance, the nasally directed eye preceded the temporally directed eye for four of six observers. Experiment  2 also confirmed that the bifixation field was smaller at near convergence distances but the nasally directed eye always lagged behind the temporally directed eye at the limits of the bifixation field. We confirmed Hering's first prediction that the bifixation field is smaller at near convergence distances than at far ones. However, the majority of our results indicate that the nasally directed eye lags behind the temporally directed eye at the limits of the bifixation field, contrary to Hering's prediction. We conclude that the eyes drift toward their tonic state of vergence when fusion breaks.

## 1. Introduction

Binocular single vision and stereoscopic depth perception are critically dependent on precise coordination of the two eyes such that the left and right eyes' images fall on corresponding or nearly corresponding points in the two eyes. Bifixation refers to the images of fixated objects falling on the foveas in both eyes. The bifixation field refers to the total area over which the two eyes can accurately bifixate a target. Establishing bifixation on a target in the binocular visual field is achieved via binocular eye movements that typically involve both a version component and a vergence component. Version refers to a binocular eye movement in which both eyes rotate by an equal amount in the same direction. Vergence eye movements are those in which the two eyes rotate by an equal amount but in opposite directions. One subset of binocular eye movements involves a pure version movement with no vergence. Such a movement would trace an arc intersecting the centers of rotation in the two eyes and the fixation point. Another subset of binocular eye movements involves a pure vergence movement with no accompanying version. These movements trace parabolic arcs intersecting the fixation point and the centers of rotation in the two eyes. All other binocular eye movements are combinations of version and vergence movements.

Tyler [1] argues that the role of vergence, in three-dimensional space perception, is similar to version eye movements in viewing a two dimensional scene. The general layout of a two dimensional scene is apparent without moving our eyes. However, we must change our gaze to foveate any details of the scene. Tyler describes the three dimensional case as follows:

The stereoscopic situation is analogous to perception of two-dimensional space with a high-acuity fovea controlled by lateral eye movements. It is clear that the general layout of objects in two-dimensional space is readily perceived across the 180 degree field without lateral eye movements…. What the eye movements accomplish is detailed perception of a chosen part of the perceived scene. It seems reasonable to suppose that vergence movements serve the same role for the third dimension, bringing regions of interest into the range of best disparity discrimination ([1] p. 283).

For Tyler, vergence is analogous to the commonly known function of version eye movements, the selection of small areas of the visual scene for detailed inspection with the fovea.

Hering [2] described the two eyes as a single mechanical and perceptual organ which he called the double eye. His theory, generally referred to as the law of equal innervation, accounts for how the eyes move together as a single mechanical organ. Hering's law is based on two putative kinds of innervation: one for the conjunctive (version) component of eye movements and one for the disjunctive (vergence) component of eye movements. Each type of innervation is equal in magnitude in the two eyes and binocular eye movements involve the pooling of the two types of innervation (see [3] for a more detailed discussion).

The pooling of version and vergence innervations has implications for the angular extent over which the two eyes can move together at different viewing distances. Specifically, the combination of version and vergence innervations relates to the change in size of the bifixation field as a function of viewing distance. To illustrate, consider the two eyes tracking a bifixated target into the periphery. At far convergence distances, where the visual axes (the visual axis is the line containing the point on which the eye is fixating, the optical nodal point of the eye, and the center of the fovea (Howard, 2012); a visual line is any straight line passing through the pupil and optical nodal point joining a distal object and a unique location on the retina) are nearly parallel (i.e., the eyes are converged at infinity), the vergence innervation is negligible relative to the version innervation. At near convergence distances, however, a large vergence innervation is required to maintain bifixation at the desired distance. Since the version and vergence innervations are congruent in the nasally directed eye and incongruent in the temporally directed eye, Hering predicted that the bifixation field should be smaller at near viewing distances, when the vergence innervation is large, than at far viewing distances, when the vergence innervation is small.

In support of his analysis above, Hering [2] made two critical observations. First, he measured the area over which each eye could move on its own by impressing a foveal afterimage on one eye when it was in primary position. Hering then moved his gaze away from primary position, attending to the afterimage. When he reached the extent to which he could move his eye in a given direction, he made a mark on a piece of glass indicating the location of the afterimage. Repeating this procedure for each eye, for near and far viewing distances, and for different directions of gaze, Hering mapped out a two-dimensional area over which he could direct the visual axis of each eye individually. The key observation was that the maximum horizontal excursion of a single eye was larger for far viewing distances than for near ones. Second, Hering reported that the area over which he could bifixate a target was relatively small compared to the combined areas over which each eye could move on its own.

Extending Hering's [2] observations and rationale, Mapp et al. [5] tested the prediction that the maximum excursion of the left eye to the right and the right eye to the left is greater at far convergence distances than for near convergence distances. Importantly, Mapp et al. noted that in such extreme eye positions, it would be impossible to bifixate the target and speculated that the laws of equal innervation may only hold within the bifixation field. They suggested that a better test of Hering's hypothesis would be to examine the width of the bifixation area as a function of distance rather than the extreme position of the eyes.

Hering [2] also posited a mechanical and a structural factor influencing the extent of the bifixation field and the relative positions of the eyes when bifixation failed. The mechanical factor was that the medial recti play a more active role in moving the eyes because they are stronger muscles than the lateral recti. This speculation was later supported experimentally by Alpern and Ellen [6], Westheimer and Mitchell [7], and Yarbus [8]. Therefore, owing to the stronger medial recti, Hering predicted that, at the limits of the bifixation field, the nasally directed eye should overshoot the temporally directed eye. The structural factor Hering referred to was the restriction of the bifixation field due to facial structures such as the nose and orbital bones occluding the bifixated target to one eye in extreme gaze positions. This is a purely geometric constraint resulting from the optics of the eye and the protrusion of the nose and orbital bones.

In summary, the goal of this paper is to test three predictions derived from Hering's law of equal innervation. The first prediction is that the bifixation field is smaller for near convergence distances than for far distances, owing to increasingly incongruent vergence and version innervations at nearer viewing distances. The second prediction is that when bifixation breaks down, the nasally directed eye precedes the temporally directed eye, owing to the superior strength of the medial recti over the lateral recti. Third, we determine which boundaries of the bifixation field are imposed by facial structures. In Experiment 1, we test the first two predictions by examining the horizontal extent of the bifixation field for two convergence distances, using a stereoscopic apparatus which allows us to record eye signature information necessary to determine the relative positions of the eyes when bifixation breaks down. In Experiment 2, we extend these measurements to a 2D area in the frontoparallel plane to examine the spatial extent of the bifixation field for three convergence distances, testing all three predictions above. In anticipation, our data support the claim that the bifixation field is smaller at near convergence distances than for far distances, consistent with Hering's prediction, but only partially support the prediction that the nasally directed eye precedes the temporally directed eye at the limits of the bifixation field. Facial structures limit the extent of the bifixation field for moderate to long viewing distances.

## 2. Experiment  1

We measured the horizontal extent over which the eyes can move together and maintain bifixation for viewing distances of 32 cm and 125 cm. Second, we ascertained the relative position of the eyes at the limits of the bifixation field.

### 2.1. Method

#### 2.1.1. Observers

There are six observers, four females and two males, from the Department of Psychology, York University participated. The observers ranged in age from 21 to 33 years with a mean age of 25.7 years. All had normal binocular vision and were experienced in psychophysical experiments. Those who required an optical correction wore contact lenses during the experiment.

#### 2.1.2. Apparatus and Stimuli

**Figure 1:**
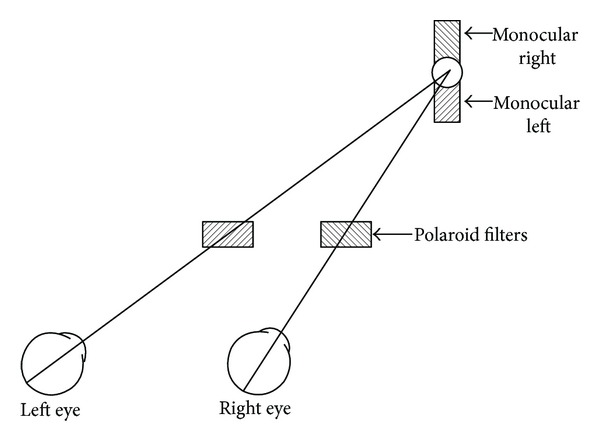
Illustration of the stimulus and the arrangement of the Polaroid filters in Experiment 1.

The apparatus consisted of a motorized boom, suspended above the observer's head such that its centre of rotation was vertically aligned with the centre of rotation of either the right or the left eye. Two light-boxes, containing the stimuli, were positioned along the boom, at eye level, at viewing distances of 32 cm and 125 cm. Each stimulus consisted of a binocular dot, 0.6 degrees in diameter, flanked above and below by Nonius lines, 2.3 degrees long by 0.3 degrees wide. The Nonius lines were made visible to the right and left eyes via polarized filters such that one filter was placed in front of one of the Nonius lines and a complimentary filter was positioned before the appropriate eye.  Figure 1 presents a schematic of the stimulus. Observers sat with their heads fixed by a bite-bar and they controlled the position of the boom via a joystick.

#### 2.1.3. Procedure

**Figure 2:**
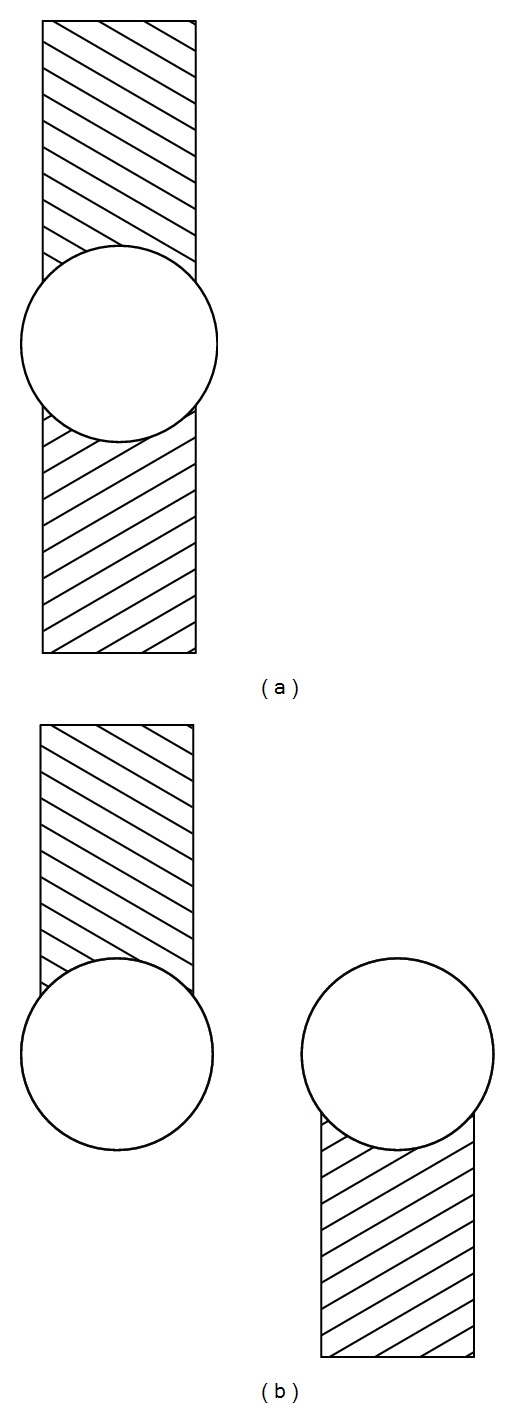
Appearance of the stimulus (a) within the bifixation field and (b) beyond the bifixation field.

The observers' task was to fixate the stimulus binocularly while either moving it away from their median plane (ascending trials) or moving it towards their median plane (descending trials) until it appeared misaligned (see Figure 2(b)) or aligned (see Figure 2(a)), respectively. The point at which the stimulus changed from appearing misaligned (aligned) to appearing aligned (misaligned) demarked the outer horizontal limit of the bifixation field. The angular position of the boom, with respect to the observers' median plane, at this limit, was recorded from a protractor attached to the boom. Additionally, the relative position of the eyes at the “breaking point” was determined by the observer's report of the position of (a) the upper half of the stimulus relative to the lower half and (b) the upper half of an afterimage induced with the eyes at the “breaking point” relative to the lower half.

Each observer performed a total of 92 trials, presented in four blocks of 23 trials each. Within each block, the limit of the horizontal bifixation field was measured 20 times at one convergence distance (32 cm or 125 cm) and one direction of gaze (rightward or leftward). Equal numbers of ascending and descending trials were presented randomly within each block and block order was randomized among observers. Three additional trials, at the end of each block, confirmed the relative position of the eyes at the “breaking point” as follows. With the observer's gaze at the limit of the bifixation field, an afterimage of the misaligned stimulus (see Figure 2(b)) was induced by a bright flash within the stimulus box. After receiving the afterimage, observers fixated a vertical line, in their median plane, and reported the position of the two components of the afterimage (the temporally and nasally directed eye's components) relative to the fixation line.

**Figure 3:**
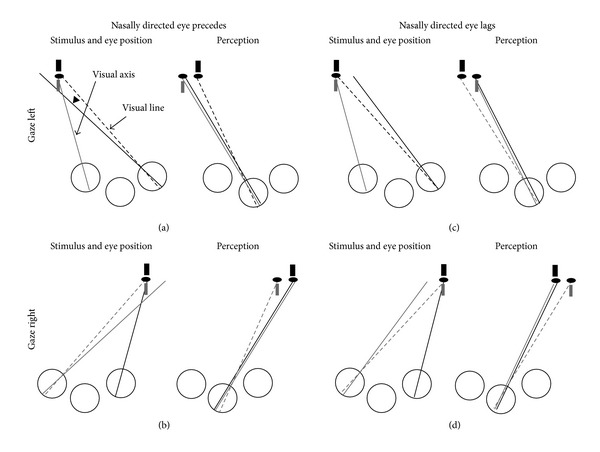
Predicted perceived Nonius/afterimage misalignments based on Wells [4] and Hering's [2] laws of visual direction. In (a) the nasally directed eye precedes the temporally directed eye with associated perception of Nonius lines or afterimages; (b) same as (a) but for rightward gaze; (c) the nasally directed eye lags the temporally directed eye in leftward gaze with associated perception of Nonius lines or afterimages; (d) same as (c) but for rightward gaze. See text for more details.

Figure 3 illustrates how the perceived relative position of the Nonius lines and afterimages indicates the relative position of the eyes when fusion breaks. The position of the stimulus and the eyes is given in the left panel of each quadrant. The associate perception is specified at the cyclopean eye in accordance with Wells [4] and Hering's [2] laws of visual direction and their modification by Mapp and Ono [9]. Stated briefly, Wells/Hering's laws state that a stimulus on the visual axes of the two eyes transfers to a common axis (cyclopean axis), which is a line passing through the intersection of the axes and a point midway between the eyes. Additionally, the angle between the visual axis and a monocular visual line transfers unaltered to the cyclopean eye. Consider the top left sector of Figure 3. The left half of this sector illustrates the stimulus and eye position and the right half illustrates the subjective perception. If in extreme leftward gaze the nasally directed right eye overshoots the temporally directed left eye, the right eye's visual axis precedes the binocular target. Therefore, the Nonius lines will appear to break such that the top (right eye's) Nonius line is to the right of the bottom (left eye's) Nonius line. The same perception is predicted if the nasally directed left eye precedes the temporally directed right eye in extreme rightward gaze, illustrated in the bottom left sector. Next, consider the top right sector. If in extreme leftward gaze the nasally directed right eye lags behind the temporally directed left eye, the right eye's visual axis lags behind the binocular target; the Nonius lines will appear to break such that the top (right eye's) Nonius line is to the left of the bottom (left eye's) Nonius line. The same perception is predicted if the nasally directed left eye lags behind the temporally directed right eye in extreme rightward gaze, illustrated in the bottom right sector.

### 2.2. Results

#### 2.2.1. Width of the Bifixation Field

**Figure 4:**
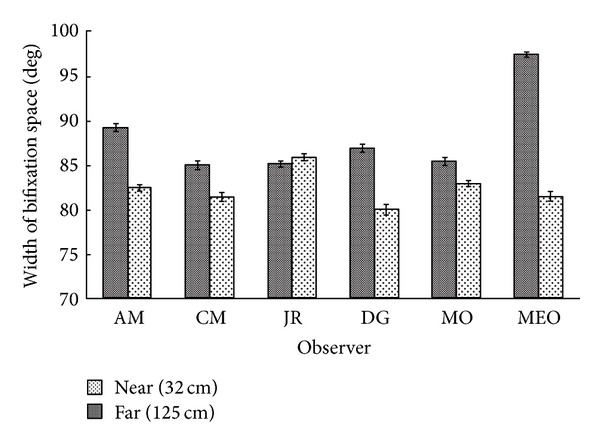
Mean width (±SEM) of the bifixation field as a function of convergence distance.

The mean width of the bifixation field, specified from the midpoint between the two eyes, was larger at 125 cm than at 32 cm for all but one observer (see Figure 4). The mean far-near difference between observers was 5.8° (SEM = 2.3°), *t*(5) = 2.49, *P* < .05,  and  *r* ^2^ = 0.55.

**Figure 5:**
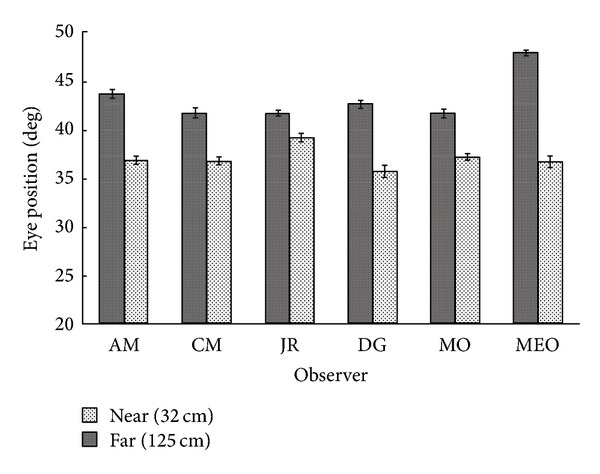
Mean position of the temporally directed eye (±SEM) at the limit of the bifixation field as a function of convergence distance.

The mean position of the temporally directed eye (i.e., left eye to the left and right eye to the right) at the “breaking point” is illustrated in Figure 5. For all six observers, the temporally directed eye rotated to a greater extent when viewing the target at 125 cm than when viewing it at 32 cm. The overall mean far-near difference was 6.1° (SEM = 1.2°), *t*(5) = 5.06, *P* < .01,  and *r* ^2^ = 0.84.

#### 2.2.2. The Relative Position of the Eyes at the Limit of the Bifixation Field

Based on the three afterimage trials at the end of each block, we determined the relative position of the eyes at the limit of the bifixation field. For all six observers, at both the 32 cm and the 125 cm convergence distances, the temporally directed eye maintained fixation on the target and the nasally directed eye broke fixation at the limit of the bifixation field. Moreover, the nasally directed eye lagged behind the fixation target for all six observers at the 32 cm convergence distance and it preceded the target for four of the six observers at the 125 cm convergence distance.

The data from Experiment 1 clearly support the prediction based on Hering's [2] law of equal innervation that the bifixation field is smaller at near viewing distances than at far viewing distances. However, the data indicating the relative position of the eyes at the limit of the bifixation field provided only partial support for Hering's prediction that the nasally directed eye precedes the temporally directed eye at the limit of the bifixation field.

The measurements in this experiment were limited to leftward and rightward eye movements in the horizontal plane of regard. In Experiment 2, we extended our investigation to match Hering's [2] measurements of the bifixation field over a 2D area.

## 3. Experiment  2

The purpose of this experiment is to address three issues about the spatial extent of the bifixation field. First, we measured the 2D area over which the eyes can move together and maintain bifixation. Second, we ascertained the relative position of the eyes at the limits of the bifixation field. Third, we determined the locations in the frontal plane where the bifixation field is limited by facial structures.

### 3.1. Method

#### 3.1.1. Observers

There are four male observers, experienced in psychophysical experiments, one author (PG) and three naïve observers participated. Observers ranged in age from 25 to 31 years with a mean age of 28.3 years. All reported normal binocular vision. Those who required an optical correction wore contact lenses during the experiment.

#### 3.1.2. Apparatus and Stimuli

Stimuli were generated on a Macintosh power PC and displayed on a polarized rear projection screen, subtending 131 degrees horizontally and 102 degrees vertically at a 57 cm viewing distance. Two projectors, one for each eye's image, were fitted with orthogonally oriented polarizing filters. A complimentary pair of polarizing filters was placed in front of the observer's eyes. Observers sat with their heads fixed in a chin and forehead rest 57 cm in front of the display. Their responses were recorded via a trackball mouse. Stimuli consisted of a binocular dot flanked above and below by Nonius lines, visible to the right and left eyes, respectively. Additionally, binocular vertical lines flanked the dot on either side, positioned 0.9 degrees on either side of the centre of the binocular dot. These binocular flanking lines operationalized a specific criterion for successful/unsuccessful bifixation.

**Figure 6:**
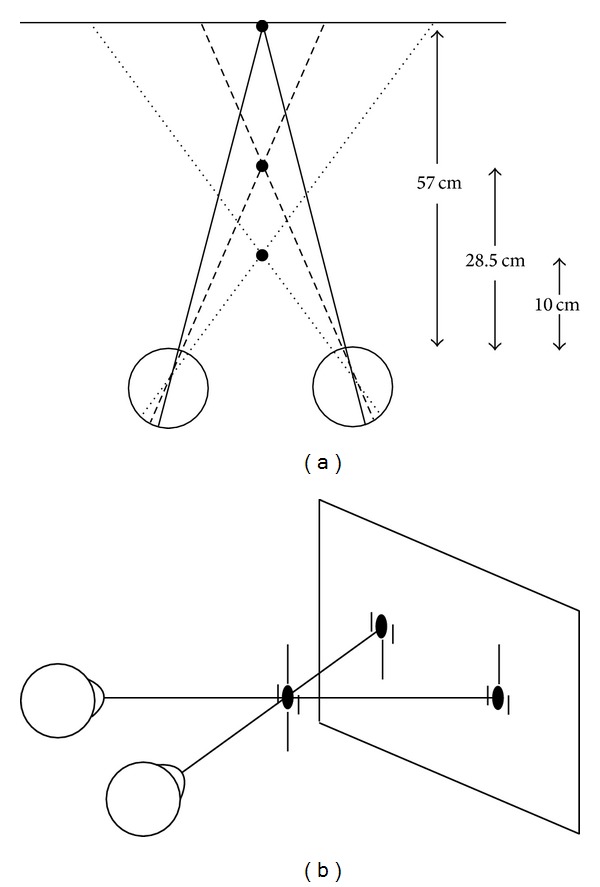
Illustration of the three vergence conditions in Experiment 1. Observers “free-fused” the fixation stimulus for the 28.5 and 10 cm distances. (a) Top view and (b) oblique view for one of the free-fused stimuli.

**Figure 7:**
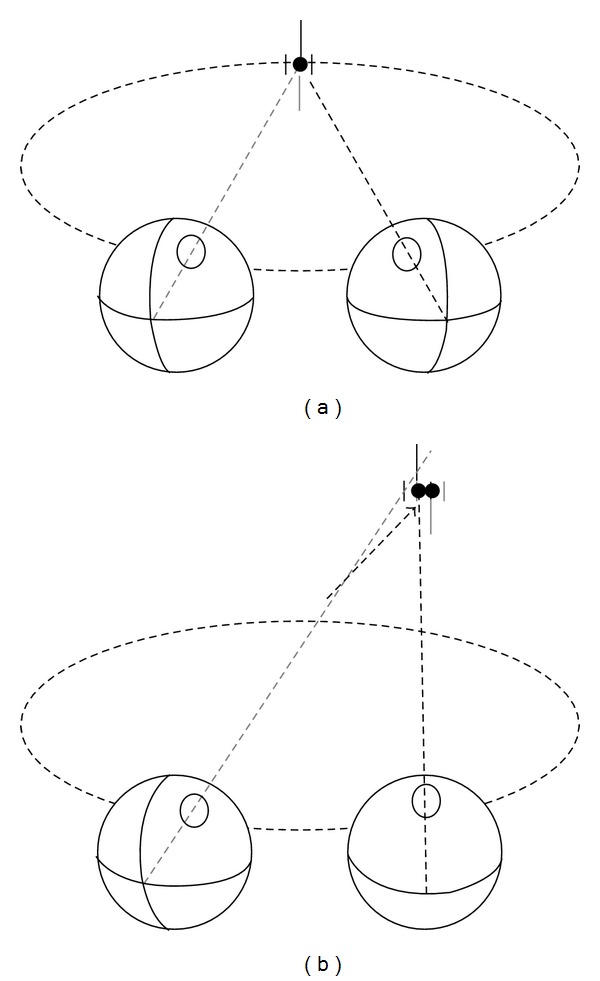
Illustration of the observers' task in Experiment 2. A typical ascending trial: after free-fusing the stimulus, observers moved it out along one of twelve radii until the Nonius lines were perceived as misaligned. Initial eye posture is shown in (a). Perception as the eyes reach the limit of the bifixation field is shown in (b). The black Nonius line was visible to the right eye; the grey Nonius line was visible to the left eye. Dashed circle represents an isovergence circle for reference.

Right and left eye images were presented at one of three separations to simulate one of three viewing distance/vergence conditions: 57 cm (6.5 degree convergence) (these values are calculated assuming an interpupilary distance of 6.5 cm), 28.5 cm (13 degree convergence), and 10 cm (36 degree convergence). In the 57 cm condition, the actual distance of the display, the fixation stimulus, and the left and right eye's Nonius lines were positioned at the centre of the screen. To simulate a 28.5 cm viewing distance, the right eye's image was displaced 3.25 cm to the left of centre and the left eye's image was displaced 3.25 cm to the right of centre. To simulate a 10 cm viewing distance, the displacement was 15.2 cm for each eye's image (see Figure 6). Observers could move the stimulus along one of 12 radii (24 radii for observer PG) in the frontoparallel plane with a track ball. Upward movements on the trackball moved the stimulus away from the center of the display; downward movements on the trackball moved the stimulus towards the center of the display. Radii were at 30-degree intervals (15-degree intervals for observer PG) converging at the centre of the display. On a given trial, observers moved the stimulus away from or towards the centre of the display along one of these radii. An example is shown in Figure 7.

#### 3.1.3. Procedure

Each trial began with the stimulus centred on the screen. For the convergence distances (28.5 and 10 cm), observers free-fused the stimulus by crossing their eyes until the binocular dot was fused and the Nonius lines were aligned. The fused stimulus was initially straight ahead of the observer. Observers then moved the stimulus slowly out from the centre along a randomly chosen radius. Observers became aware of which radius they were providing a measurement for after they moved the target. For the 57 cm viewing distance, the right and left eye's images were not separated; the observer simply fixated the binocular dot and moved the stimulus out from the centre.

Experimental runs were blocked according to convergence distance (10, 28.5, 57 cm) and the direction of adjustment (ascending/descending). Block order of convergence distance was varied across observers. Ascending and descending adjustment blocks alternated on each block of the experiment. On a given run in an ascending trial, observers moved the fused stimulus outward until the binocular dot appeared diplopic. Observers were instructed to move the stimulus out to the point where the Nonius lines became misaligned such that one of the Nonius lines was collinear with one of the flanking binocular lines. He then pressed the mouse button indicating that location. In a given descending trial, the observer moved the stimulus outward until it was clearly diplopic and then moved it back towards the centre until the Nonius lines were subjectively aligned and indicated the position of the stimulus with a mouse click. All trials began with the stimulus straight ahead in order to allow observers to first free-fuse the stimulus and move it to the appropriate position for testing.

In all trials, observers indicated the relative positions of the Nonius lines when fusion broke by selecting one of three options presented on the display at the end of each trial. Observers reported whether the right eye's (top) Nonius line appeared (a) to the left of the left eye's (bottom) Nonius line, indicating that the nasally directed eye* lagged* behind the temporally directed eye, or (b) to the right of the left eye's Nonius line, indicating that the nasally directed eye* preceded* the temporally directed eye; (c) some part of the stimulus was occluded by part of the observer's face or border of the display. Figure 3 illustrates the relationship between perceived misalignment of the Nonius lines and the relative position of the eyes. Observers YF, SN, and NG completed 12 sessions (six in each direction: from single to double or double to single), each containing 36 trials for a total of 432 trials for each of these participants. In a given session, observers viewed each stimulus permutation once (three vergence conditions X 12 radii). Observer PG completed 12 sessions (six in each direction), each containing 72 trials for a total of 864 trials (three vergence conditions X 24 radii).

### 3.2. Results

#### 3.2.1. Polar Axes

**Figure 8:**
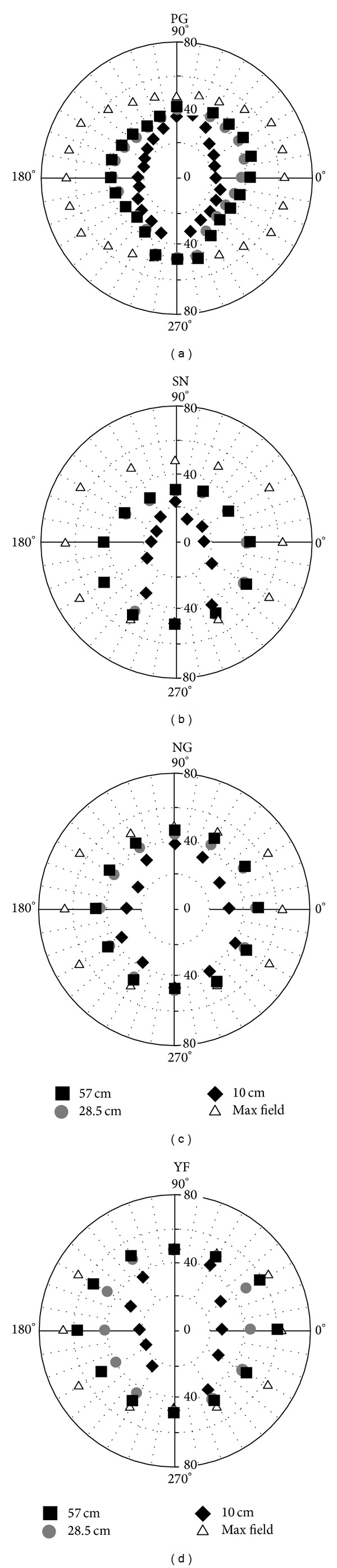
Individual polar plots of mean settings for “single to double” and “double to single” adjustments along each radius for three convergence distances. Closed symbols represent each simulated convergence distance. “Max field” refers to the extent of the stimulus display.

Individual polar plots of the mean settings based on 12 observations, six for “single to double adjustments” and six for “double to single adjustments”, along each radius are illustrated in Figure 8. Closed squares represent mean adjustments for the 57 cm fixation distance, gray circles for the 28.5 cm convergence distance, and the black diamonds represent the 10 cm convergence distance condition. Open triangles denote the extent of the stimulus display and they are presented to indicate where, if ever, the bifixation field was limited by the dimensions of the display. This happened most frequently for the 270-degree radii and the two-adjacent radii. It is apparent from concentric distribution of the data as a function of convergence distance, for each of the four observers, that the angular extent of the bifixation field shrinks as the convergence distance is reduced. Moreover, the shape of the bifixation field at different convergence distances is defined by the distribution of the different data symbols. This is most clearly defined for observer PG, who made observations on a larger sample of radii.

#### 3.2.2. Cartesian Axes

**Figure 9:**
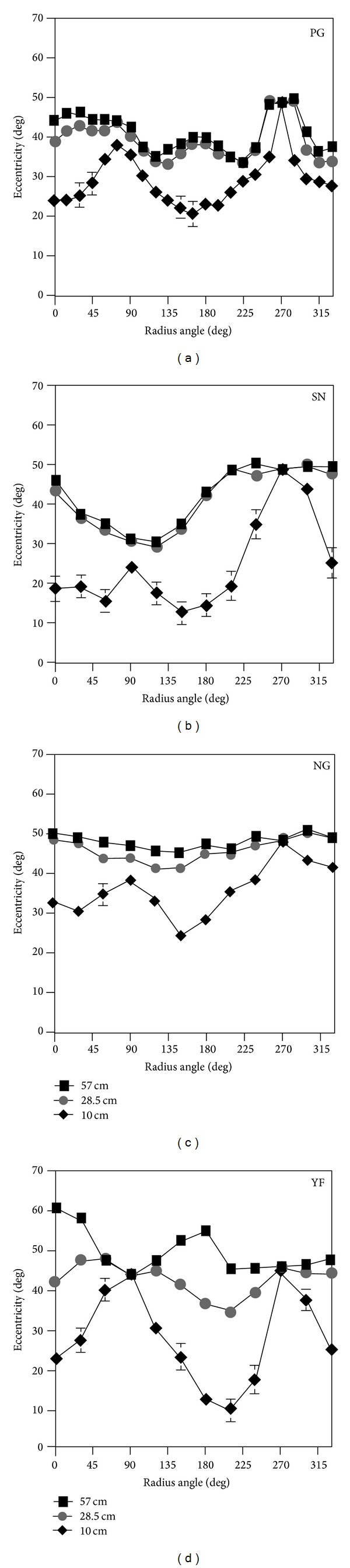
Individual Cartesian plots of the mean settings for “single to double” and “double to single” adjustments along each radius. Error bars represent ±1 SEM.

Individual Cartesian plots are illustrated in Figure 9. These plots are provided to illustrate both the variability of the data (error bars = ±SEM) and the radii for which differences in the angular extent of the bifixation field for different simulated convergence distances are at a maximum and minimum. It is clear from the graphs that the differences in the extent of the bifixation field are greatest at 0 and 180 degrees or along the horizontal meridian for each of the observers. The width of the bifixation field is clearly smaller at the 10 cm convergence distance than the 28.5 or 57 cm distances. The difference between the 28.5 and 57 cm distances are apparent for two observers (PG, YF) at 0 and 180 degrees, but less so for SN and NG. These data are consistent with the predictions based on Hering's [2] law of equal innervation. It is not surprising that the extent of the bifixation field converges for the three fixation distances at the 90-degree and 270-degree radii. Conjugate up/down eye movements have no version component and have a relatively constant vergence component.

#### 3.2.3. Nonius Data

**Figure 10:**
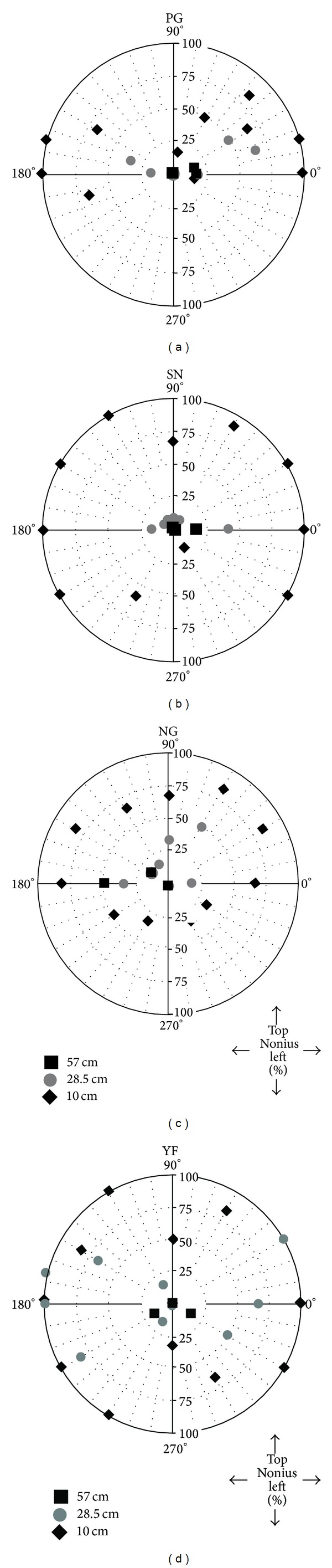
Individual polar plots indicating the percentage of trials; the Nonius lines appeared misaligned with the top line displaced towards the left for each radius. Percentage is plotted along vertical radii; radius angles are plotted around the circumference.

We next tabulated the percentage of trials which observers reported that the top Nonius line was to the left of the bottom Nonius line along each radius for each convergence distance. These data are illustrated on Polar axes in Figure 10. Each radius in these plots represents judgments made along each radius in the experiment. The distance away from the origin represents the percentage of trials which observers reported that the top Nonius line was seen to the left of the bottom Nonius line, indicating that the nasally directed eye lagged behind the temporally directed eye.

Despite being given a choice of three responses to describe their perception of the bifixation stimulus at the limit of the bifixation field, observers never reported that the top Nonius line was seen to the right of the bottom Nonius line when fusion broke. Importantly, this indicates that the nasally directed eye never preceded the temporally directed eye in extreme binocular gaze positions for any of the convergence distances tested in this experiment, contrary to Hering's [2] prediction.

Inspecting the Nonius data more closely reveals another pattern. At the 57 cm convergence distance, with few exceptions, observers reported that the Nonius lines remained aligned until the stimulus was occluded to one eye. Trials for which the Nonius stimuli became misaligned before the stimulus was partially occluded to one eye, for this convergence distance, involved a near horizontal movement of the eyes, a radius close to 0 or 180 degrees. Nonius misalignment was reported at the 57 cm viewing distance along the 0-degree radius (an extreme rightward gaze) for observers PG and SN. Observers YF and NG reported Nonius misalignment for near extreme leftward movements of the eyes (210-degree radius for YF; 180-degree radius for NG). In the 10 cm convergence condition, however, the Nonius stimuli became misaligned before the stimulus was partially occluded to one eye in nearly all trials. The restriction of the bifixation field was most pronounced along radii close to 0 and 180 degrees. The 28.5 cm convergence condition yielded intermediate results with Nonius misalignment observed for near horizontal gazes and oblique gazes along radii close to horizontal.

Observers reported fewer incidences of Nonius misalignment for near vertical eye movements, involving radii close to 90 and 270 degrees. In fact, the limiting factor along the 270-degree radius was the size of the display. For this radius, all four observers were able to accurately track the target and maintain subjective Nonius alignment until it reached the edge of the display. Additionally, all four observers' data show a reduction in the percentage of “top Nonius left” responses near the 90-degree radius in the near vergence condition. For this and adjacent radii (particularly for observer PG) the stimulus was partially occluded by the observers' orbital bones or the top of the display.

**Figure 11:**
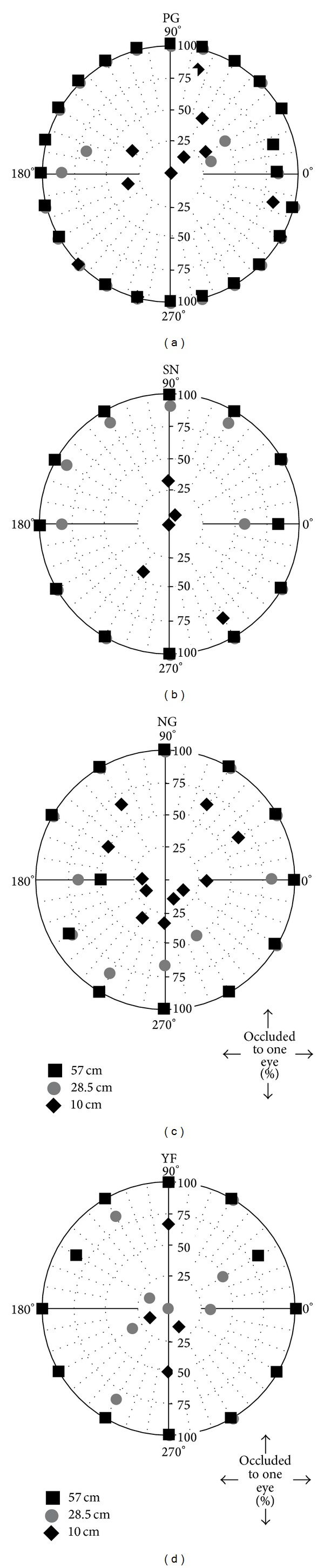
Individual polar plots indicating the percentage of trials; the fixation stimulus became occluded to one eye. Percentage is plotted along vertical radii; radius angles are plotted on the circumference.

The Nonius data are replotted in Figure 11 to illustrate the percentage of trials which observers reported; one eye's image became occluded by a facial structure in extreme gaze. When the viewing distance was 57 cm, observers were able to track the stimulus and maintain Nonius alignment until one eye's image was occluded by a facial structure on nearly 100% of the trials. This is illustrated by the closed squares distributed around the circumference of the graph for all observers. At the 28.5 cm viewing distance, observers were still able to track the stimulus to the point where one eye's image was occluded by a facial structure in many trials. This is illustrated by the distribution of gray circles near the outer edge of the polar graph. At the 10 cm viewing distance, however, all observers' bifixation broke down before the target reached the occlusion limits of the bifixation field. This is illustrated by the clustering of black diamonds near the centre of the polar graph. Observers were able to track the stimulus farther for near vertical eye movements, however, as illustrated by the scatter of closed diamonds along the 90 and 270 degree radii (and adjacent radii as well).

#### 3.2.4. Statistical Analysis

The concentric distribution of the data points for the three viewing distances in Figure 8 indicates that the area of the bifixation field shrinks with near viewing distances. Figure 9, in addition, illustrates that the vertical extent of the bifixation field is nearly equal at all three viewing distances. This fits with Hering's [2] hypothesis because there is no version component and a fairly constant vergence component for up/down eye movements in the median plane. Figure 11 indicates that occlusion of one eye's image by facial structures was the limiting factor restricting the bifixation field for eye movements along radii on either side and including the 90-degree and 270-degree radii. Therefore, we performed paired *t*-tests on the individual data for near horizontal eye movements, using the overall mean of the 0, 30, 150, 180, 210, and 330-degree radii for observers SN, NG, and YF. For observer PG, used the mean of the 0, 15, 165, 180, 195, and 345-degree radii. We tested the differences in widths of the bifixation field between 57 and 28.5 cm and between 28.5 and 10 cm. This analysis revealed that the width of the bifixation field at 28.5 cm was significantly smaller than at 57 cm, *P* < 0.001, and the bifixation field at 10 cm was significantly smaller than at 28.5 cm, *P* < 0.001, for all observers. Lastly, it is statistically improbable that the settings of the three convergence distances be identically ranked for all four observers by chance. This was verified by Friedman's two-way analysis of variance by ranks, *P* < 0.05.

This experiment confirmed Hering's [2] prediction that the area over which the two eyes can move in concert, bifixating a target, is restricted both by viewing distance and the facial structures of the observer. Specifically, at short simulated viewing distances the bifixation field was markedly reduced relative to larger viewing distances. However, we found no support for the prediction that the nasally directed eye precedes the temporally directed eye at the limit of the bifixation field. We discuss the possible reason for this pattern of results in the next section.

## 4. Discussion

The first finding of this study was that the bifixation field is smaller for near viewing distances than for far. Experiment 1 showed this for measurements in the horizontal plane of regard. Experiment 2 expanded these measurements to map a 2D bifixation area similar to Hering's [2] mapping. Together, the experiments provide strong support for the first prediction based on the law of equal innervation.

The second finding was that, for most of our measurements, the relative position of the two eyes, at the limits of the bifixation field, was opposite to what Hering [2] predicted. In the near convergence distance condition (32 cm) of Experiment 1, all six participants reported that the nasally directed eye lagged the temporally directed eye, at the limit of the bifixation field, opposite to Hering's prediction. In the far condition (125 cm), two of the six participants reported that the nasally directed eye lagged the temporally directed eye, opposite to Hering, and four reported that the nasally directed eye preceded the temporally directed eye, consistent with Hering. In Experiment 2, all reports of the relative position of the eyes at the limit of the bifixation field indicated that the temporally directed eye lagged the nasally directed eye. One possible reason for the nasally directed eye exclusively lagging the temporally directed eye in Experiment 2 is that the convergence distances spanned a relatively smaller range (10–57 cm). It is known that the typical resting vergence state of the eyes is approximately 1.2 m [10, 11], but there are considerable individual differences [12]. Therefore, it is possible that the eyes approach a resting state of vergence when fusion breaks at the limit of the bifixation field. This analysis is consistent with our results. The eyes diverged when fusion broke at the limit of the bifixation field in the near condition in Experiment 1 and all the convergence distances in Experiment 2. That four of six observers' nasally directed eye preceded the temporally directed eye in the far condition of Experiment 1 is likely due to the fact that this viewing distance is close to the typical resting vergence distance. That is, the resting vergence distances may have been closer than 125 cm for four of the observers, but farther than 125 cm for the other two.

The possibility that subjects' eyes drifted towards their resting vergence posture at the limits of the bifixation field is consistent with Maddox's theory of vergence eye movements [13] (we thank an anonymous reviewer for pointing this out). Maddox regarded vergence eye movements as having four additive components. The first component, he called tonic vergence, is similar in meaning to dark vergence or resting vergence as we have used above. Tonic vergence depends on the state of balanced tension in the rectus muscles and the spontaneous activity in the vergence control centre. Maddox's second component was reflex convergence which responds automatically to binocular disparity to correct any error between the to be fixated object and the intersection of the visual axes. The third component is accommodative-convergence, which is driven by the accommodative effort to maintain image sharpness. Contemporary research has shown that accommodation and convergence are dependent on one another as well as pupil diameter, known as the near triad [14]. Lastly, Maddox referred to voluntary convergence which was driven by the viewer's knowledge that a to be fixated object was nearer than the current point of intersection of the visual axes.

Two of Maddox's components are relevant to this report and we will address them in turn. As stated above, Maddox's concept of tonic vergence anticipated the modern concept of resting vergence or dark vergence, that is, the vergence state of the eyes in the absence of any fusible stimulus. One contemporary study by Francis and Owens [12] investigated dark vergence and showed that vergence drifted toward the dark vergence posture when the fusional stimulus was presented in the periphery but with the eyes converged on a central location. Specifically, when the peripheral fusional target was nearer in depth than the individual's dark vergence posture, convergence was beyond the target distance; when the fusional target was farther in depth than the individual's dark vergence distance, convergence was nearer than the target distance. Their report is consistent with our interpretation of the data reported here. Indeed, contemporary research, based on Maddox's theory, predicts the pattern of results we report here and would further predict that variations in the extent of the bifixation field as a function of viewing distance depends on the individual's dark vergence distance. To test this prediction, future experiments might measure the bifixation field for each participant at their dark vergence distance compared with nearer and farther viewing distances.

Considering the link between accommodation and vergence, Maddox's third component, the synergistic combination of accommodation, and convergence eye movements at near distances would be reduced or absent in older individuals with presbyopia (Herring law predicts that reduced accommodative vergence, resulting from presbyopia, would lead to an expansion, not reduction, of the bifixation field at near distances. Hering argued that the field is larger at far distances because the vergence component is smaller. Extending that logic to a weaker vergence component at near viewing distances, which is what happens with presbyopia, predicts that the bifixation field should be larger for older subjects relative to healthy young subjects). However, the participants in this study were significantly younger (maximum age: 33 years) than the typical onset age (40 years or older) of presbyopia and so we discount this as a contributing factor in our experiments. In Experiment 1, observers fixated on a real target presented at one of two physical viewing distances but in Experiment 2 the stimuli were at the same physical distance (57 cm) in all conditions and the observers converged to the “virtual” distances of 28.5 and 10 cm, resulting in accommodation/vergence mismatch. We believe this mismatch is small, however, for at least two reasons. First, reduction of the bifixation field at the near viewing distances was observed in both experiments though the discrepancy between accommodation and vergence was only present in Experiment 2. Second, Owens and Leibowitz [15] reported that a fixation point is not an optimal accommodative stimulus. They reported that the accommodative response to a small luminous disk fixation stimulus is highly correlated with the individual's resting focus rather than the distance of the disc. Therefore, our fixation stimulus may not elicit a strong accommodation response and therefore the discrepancy between accommodation and vergence in Experiment 2 was small. We conclude that, in the context of the present study, accommodative convergence had a minimal impact on our results.

In addition to testing the specific predictions of Hering's [2] hypothesis, our data have practical implications for 3D media and virtual reality. The premise of a 3D display is that observers can be immersed in a virtual 3D environment defined mostly by binocular disparity. A major problem facing designers of this technology is the fact that users become fatigued and experience discomfort after prolonged use. Our data serve as another resource for display engineers requiring knowledge of the biological system when setting the parameters (such as display size and viewing distance) of their 3D systems.

The ability to discriminate the relative depth between two targets is the best when one of the targets is fixated. If an observer fixates a point, with symmetrical vergence, for example, their ability to discriminate small depth intervals deteriorates as the target stimuli are moved to eccentric locations away from the fovea. Ogle [16] showed that as targets with relative disparity were moved away from the fovea along the horizontal meridian, stereoacuity was degraded. Tyler [1] argues that this decrease in stereoacuity would occur if targets were moved out along any meridian (horizontal, vertical, or oblique) and is presumably a function of the increase in receptive field size with eccentricity.

Consider how removing a target display from the plane of fixation along an axis perpendicular to the frontoparallel plane affects our ability to discriminate small depth intervals within the display. Blakemore [17] varied the magnitude of the depth pedestal (absolute disparity or disparity between the two slit targets and the fixation point) of two slit targets such that they were positioned in front of or behind the fixation plane on any given trial. He found that stereothresholds increased as the discrimination targets were positioned further in front of the fixation point (crossed absolute disparity) or behind the fixation point (uncrossed absolute disparity). The angle of convergence was constant in Blakemore's study and the depth pedestal (absolute disparity) was introduced by horizontally shifting the stereo half images relative to the fixation point. Presumably, an equivalent degradation in stereoacuity would result from absolute disparities or depth pedestals, introduced by misconverging in front of or behind the stimulus plane. Therefore, for optimal stereoscopic performance, stimuli should be positioned within the bifixation field to ensure that the targets are accurately fixated and absolute disparity is minimised.

## 5. Conclusion

We have presented data from two experiments supporting the prediction, from Hering's law of equal innervation that the bifixation field is smaller at near convergence distances than at far convergence distances. On the whole, our data are inconsistent with the prediction that the nasally directed eye precedes the temporally directed eye at the limit of the bifixation field, owing to its greater strength and the incongruent version and vergence components, present in the temporally directed eye. Our data are more consistent with the idea that the eyes drift towards their resting state when fusion breaks. At farther viewing distances, facial structures such as the nose and orbital bones contribute more to the restriction of the bifixation field than the differences in the component innervations. Lastly, since stereopsis is dependent on well-calibrated and precise vergence, a mapping of the bifixation field can inform 3D display engineers as they develop new displays that fill more and more of the visual field to ensure optimal performance and comfort.
